# Effects of Corn Fermented Protein as a Primary Protein Source on Growth Performance, Feed Utilization Efficiency, Hemolymph Biochemical Parameters, and Physiological and Digestive Gene Expression of Pacific White Shrimp (*Litopenaeus vannamei*)

**DOI:** 10.1155/anu/7696899

**Published:** 2026-02-04

**Authors:** Khanh Q. Nguyen, Cristhian San Andres, Adela N. Araujo, Trenton L. Corby, Melanie A. Rhodes, Scott Tilton, Timothy J. Bruce, D. Allen Davis

**Affiliations:** ^1^ School of Fisheries, Aquaculture and Aquatic Sciences, Auburn University, Auburn, 36849, Alabama, USA, auburn.edu; ^2^ The Andersons Inc., Maumee, Ohio, USA

**Keywords:** broken-line regression, corn fermented protein, gene expression, hemolymph, *Litopenaeus vannamei*

## Abstract

This study aimed to determine the effect of replacing fish meal (FM), soybean meal (SBM), and corn protein concentrate (CPC) in an equal ratio with corn fermented protein (CFP) on Pacific white shrimp, *Litopenaeus vannamei* growth, feed utilization efficiency, hemolymph parameters, and physiological gene expression. A green water recirculation system was employed with a stocking density of 30 shrimp per tank (~35 shrimp/m^2^) and an initial weight of 0.216 ± 0.007 g (mean ± standard deviation). Six experimental diets were investigated over 8 weeks. The primary protein sources in the basal diet were systematically replaced (0% – 25% diet) with CFP. No significant changes in shrimp growth indicators were found between shrimp reared on the various diets (*p*  > 0.05). We found few significant differences in feed utilization efficiency (*p*  > 0.05), but especially for feed conversion ratio (FCR; *p* = 0.372) shifts in net phosphorus retention (PR; *p*  < 0.001) were significant. Physiological gene expression analysis revealed no signs of gut inflammation or digestive enzyme disorders (*p*  > 0.05). At the same time, the hemolymph index exhibited similar patterns with no statistically significant outcome (*p*  > 0.05). The results showed that, under a setting with natural productivity, different replacement levels did not impair the growth response, feed consumption, physiological gene expression, and hemolymph indicators of Pacific white shrimp when appropriately balanced with other protein sources. The results demonstrated the feasibility of replacing the primary protein source with CFP in practical feed applications. Overall, using CFP in shrimp feed formulation resulted in a good growth rate with no adverse effects.

## 1. Introduction

The formulation of economical and practical diets for Pacific white shrimp (*Litopenaeus vannamei*) is crucial for enhancing profitability while satisfying the nutritional requirements of this rapidly expanding species. Fish meal (FM) has been recognized as one of the main animal‐based protein sources in aquaculture diets because of its high protein content, well‐balanced amino acid (AA) and fatty acid profiles, and excellent palatability [1, 2]. However, due to limitations in supply, rising demand, increased prices, and ethical concerns, the average inclusion rate of FM in shrimp diets is being reduced [3, 4]. As a result, alternative protein sources, especially plant‐based components, have garnered considerable interest [1].

Due to its high protein content, digestibility, and favorable AA profile, soybean meal (SBM) is a popular plant protein source in shrimp diets [5–7]. Yet, SBM has reduced levels of important AAs, particularly methionine, and includes antinutritional factors (ANFs), which various processes may mitigate at an increased expense [8–10]. High‐protein plant‐based components, such as corn‐derived products, have emerged as viable alternatives thanks to their nutritional density, complementing other protein sources like SBM.

Corn‐derived components like corn gluten meal (CGM) and corn protein concentrate (CPC) are by‐products of the wet‐milling process and are progressively used in shrimp feed [11, 12]. CGM has around 60% protein and reduced ANFs; however, it has lower concentrations of AAs, including arginine and lysine, compared to FM [13, 14]. CPC, a relatively new ingredient with a protein concentration of about 75%, is created by the enzymatic solubilization of corn proteins during the wet‐milling process [15]. CPC has successfully substituted up to 12% of FM in shrimp diets without adversely affecting growth or feed conversion rates [11, 15]. Nevertheless, lower growth performance was reported in diets that contained CGM and CPC at high inclusion levels, presumably due to decreased feed intake, digestibility, and deficiencies in key AAs [16, 17].

The enhancement of starch‐to‐ethanol conversion has resulted in progress in ethanol production, resulting in the creation of nutrient‐rich corn‐based coproducts, such as distillers dried grains (DDGS) and corn fermented protein (CFP). DDGS is a recognized coproduct of dry‐grind ethanol production, generally including 25%–30% crude protein (CP), 6%–9% crude fat, and under 14% crude fiber. DDGS variations include de‐oiled DDGS, characterized by a fat content below 3%, and high‐protein DDG (HP‐DDG), which has 36%–48% CP due to the extraction of fiber and oil components [18, 19]. Recent advancements in ethanol processing have led to the development of CFP, an innovative, high‐protein corn coproduct with a CP concentration of over 48% and a significantly lower oil content ranging from 3% to 8%. In contrast to traditional DDGS, CFP is subjected to mechanical separation to exclude fiber and oil, thereby concentrating residual grain and spent yeast proteins resulting in a unique nutritional profile [18]. In contrast to CPC, which has the highest CP concentration (>67%) and minimal oil content (0.5%), CFP offers a balanced protein alternative but possesses a comparatively low lysine level, requiring supplementing with crystalline AAs potentially for optimal shrimp growth [20].

Prior research has effectively decreased the incorporation of FM, CPC, or solvent‐extracted SBM in the diets of Pacific white shrimp under a range of culture conditions [20–22]. Nevertheless, research on substituting primary protein sources with CFP in practical feed, emphasizing both growth performance and animal health, is still limited. This research seeks to assess the effectiveness of CFP as a substitute for FM, SBM, and CPC in the practical diets of Pacific white shrimp in a practical green water system.

## 2. Materials and Methodology

### 2.1. Postlarvae (PLs) Nursing

PLs were obtained from Homegrown Shrimp LLC., USA (Indiantown, FL, USA). Following acclimation procedures, PLs were transferred to nursery tanks and cultured until they achieved a mean weight of 0.216 ± 0.007 g (mean ± standard deviation). Throughout the nursery phase, animals were provided commercial diets consisting of PL Raceway Plus 1, 2, 3 and PL Raceway 40–9 (Zeigler Bros, Inc., Gardners, PA, USA). Weekly weight monitoring was conducted using a subsample of 60 PLs per tank, with the resulting average weight data utilized to adjust feeding rates accordingly.

### 2.2. Diet Preparation

Six experimental diets were prepared on an iso‐nitrogenous and iso‐lipidic basis (35 g/100 g protein and 8 g/100 g lipid), of which five contained varying inclusion levels of CFP. The basal diet (Diet 1) consisted of 50 g/100 g solvent‐extracted SBM (Bunge, St. Louis, MO, USA), 6 g/100 g menhaden FM (Special Select Menhaden FM, Omega Protein Inc., Houston, TX, USA), and 7.5 g/100 g CPC (Empyreal 75, Cargill Corn Milling, Cargill Inc., Blair, NE, USA). These ingredients were subsequently substituted on an equivalent protein and ratio basis to create five additional diets incorporating 5, 10, 15, 20, and 25 g/100 g of CFP (ANDVantage 50Y, The Andersons, Inc., Maumee, OH, USA).

Proximate composition and AA profiles of primary protein sources (Table 1) and experimental diets (Tables 2 and 3) were determined at the University of Missouri Agriculture Experiment Station Chemical Laboratories (Columbia, MO, USA). Diet preparation was conducted at the Aquatic Animal Nutrition Laboratory within the School of Fisheries, Aquaculture, and Aquatic Sciences, Auburn University (Auburn, AL, USA), according to established laboratory protocols. Preground dry components and oil were measured and blended for 15 min using a Globe 20 Quart Planetary Mixer model SP20 (Globe Food Equipment Co., Dayton, OH, USA). Hot water (30%–45% by weight) was incorporated to achieve appropriate pelletizing consistency. The diet mixtures were pressure‐pelleted through a 3‐mm die using a meat grinder. Subsequently, pelleted strands were placed on wire trays and dried in a forced air oven (<45°C) overnight to reach a target moisture content below 10%. Dried pellets were then crumbled, packaged, and stored at 4°C until use.

**Table 1:** Proximate and amino acids composition (g/100 g as is) of various protein sources used to assess shrimp growth in the green water recirculating system.

Parameters	SE‐SBM	CFP	CPC	FM
Proximate composition (g/100 g)^a^				
Crude protein	43.28	49.79	77.46	66
Moisture	13.32	7.57	7.11	6.14
Crude fat	0.26	8.17	3.13	8.58
Crude fiber	3.65	7.63	0.84	0.8
Ash	5.92	3.08	1.53	19.95
Amino acids composition (g/100 g)^a^				
Alanine	1.88	3.7	6.92	4.06
Arginine	3.08	2.24	2.46	3.79
Aspartic acid	4.80	3.39	4.69	5.51
Cysteine	0.61	1.05	1.49	0.54
Glutamic acid	7.84	8.96	17.69	7.94
Glycine	1.84	1.87	2.16	4.96
Histidine	1.12	1.47	1.64	1.52
Hydroxylysine	0.04	0.02	0.06	0.25
Hydroxyproline	0.11	0.12	0	1.14
Isoleucine	2.05	2.1	3.36	2.57
Leucine	3.41	6.27	13.03	4.3
Lysine	2.81	1.79	1.31	4.74
Methionine	0.60	1.22	2.11	1.67
Phenylalanine	2.27	2.61	5.03	2.54
Proline	2.11	4.03	7.21	3.51
Serine	1.79	2.41	3.99	2.17
Taurine	0.11	0.06	0	0.69
Threonine	1.63	1.91	2.63	2.49
Tryptophan	0.55	0.39	0.48	0.56
Tyrosine	1.67	2.05	4.05	1.88
Valine	2.13	2.62	3.61	3.01
Total	42.49	50.31	83.96	59.93

*Note*: CFP, corn fermented protein ANDVantage 50Y (The Andersons, Inc., Maumee, OH, USA); SE‐SBM, solvent‐extracted soybean meal (Bunge, St. Louis, MO, USA).

^a^Proximate analysis performed by University of Missouri Laboratory (Colombia, MO, USA) with results expressed as g/100 g.

**Table 2:** Diet composition (g/100 g as is) for experimental feeds formulated on an iso‐nitrogenous and iso‐lipidic basis (35 g/100 g protein and 8 g/100 g lipid) for Pacific white shrimp (*Litopenaeus vannamei*) reared in green water recirculating systems over an 8‐week period.

Composition	Basal	CFP 5%	CFP 10%	CFP 15%	CFP 20%	CFP 25%
Fish meal^a^	6.00	4.80	3.60	2.40	1.20	—
SBM^b^	50.50	49.30	48.10	46.90	45.70	44.50
CPC^c^	7.50	6.00	4.50	3.00	1.50	0.0
CFP^d^	—	5.00	10.00	15.00	20.00	25.00
Menhaden fish oil^e^	2.50	2.50	2.50	2.50	2.50	2.50
Soy oil	3.64	3.39	3.14	2.90	2.65	2.40
Lecithin (soy)^f^	1.00	1.00	1.00	1.00	1.00	1.00
Cholesterol	0.12	0.12	0.12	0.12	0.12	0.12
Corn starch	4.34	3.49	2.64	1.78	0.93	0.08
Whole wheat	19.30	19.30	19.30	19.30	19.30	19.30
Mineral premix^g^	0.50	0.50	0.50	0.50	0.50	0.50
Vitamin premix^h^	1.80	1.80	1.80	1.80	1.80	1.80
Choline chloride^i^	0.20	0.20	0.20	0.20	0.20	0.20
Rovimix Stay‐C 35%^j^	0.10	0.10	0.10	0.10	0.10	0.10
CaP‐dibasic^k^	2.50	2.50	2.50	2.50	2.50	2.50
Proximate composition (g/100 g as is)^l^						
Crude protein	37.80	36.8	36	36.4	36.1	36
Moisture	7.29	6.93	8.98	7.38	7.23	8.03
Crude fat	8.23	8.14	7.88	8.28	8.32	8.17
Crude fiber	4.60	6.7	7	5.7	8.6	7.8
Ash	7.38	7.08	6.62	6.46	6.1	5.85

*Note:* Shrimp were fed varying inclusion levels of corn fermented protein (CFP), maintained at a stocking density of 30 individuals per tank, with an initial mean weight of 0.216 ± 0.007 g (mean ± standard deviation).

^a^Special Select Menhaden fish meal, Omega Protein Inc., Houston, TX, USA.

^b^Solvent‐extracted soybean meal, Bunge, St. Louis, MO, USA.

^c^Corn protein concentrate, Empyreal 75, Cargill Corn Milling, Cargill Inc., Blair, NE, USA.

^d^Corn fermented protein, ANDVantage 50Y (The Andersons, Inc., Maumee, OH, USA).

^e^Omega Protein Inc., Houston, TX, USA.

^f^The Solae Company, St. Louis, USA.

^g^Trace mineral premix (g/100 g premix): cobalt chloride, 0.004; cupric sulfate pentahydrate, 0.550; ferrous sulfate, 2.000; magnesium sulfate anhydrous, 13.862; manganese sulfate monohydrate, 0.650; potassium iodide, 0.067; sodium selenite, 0.010; zinc sulfate heptahydrate, 13.193; alpha‐cellulose, 69.664.

^h^Vitamin premix (g/kg premix): thiamin HCl, 4.95; riboflavin, 3.83; pyridoxine HCl, 4.00; Ca‐pantothenate, 10.00; nicotinic acid, 10.00; biotin, 0.50; folic acid, 4.00; cyanocobalamin, 0.05; inositol, 25.00; vitamin A acetate (500,000 IU/g), 0.32; vitamin D3 (1,000,000 IU/g), 80.00; menadione, 0.50; alpha‐cellulose, 856.81.

^i^MO Biomedicals Inc., Solon, OH, USA.

^j^Stay C (L‐ascorbyl‐2‐polyphosphate 35% Active C), Roche Vitamins Inc., Parsippany, NJ, USA.

^k^Acros Organics B. V. B. A. Thermo Fisher Scientific, Waltham, MA, USA.

^l^Proximate analysis performed by Midwest Laboratories Inc. (Omaha, NE, USA) with results expressed as g/100 g of feed as is, unless otherwise indicate.

**Table 3:** Amino acid profile (g/100 g as is) for experimental feeds formulated on an iso‐nitrogenous and iso‐lipidic basis (35 g/100 g protein and 8 g/100 g lipid) for Pacific white shrimp (*Litopenaeus vannamei*) reared in green water recirculating systems over an 8‐week period.

Amino acids	Basal	CFP 5%	CFP 10%	CFP 15%	CFP 20%	CFP 25%
Alanine	1.99	1.88	1.85	1.93	1.92	1.88
Arginine	2.28	2.26	2.21	2.26	2.24	2.19
Aspartic acid	3.55	3.48	3.38	3.41	3.38	3.27
Cysteine	0.51	0.52	0.53	0.55	0.58	0.56
Glutamic acid	7.38	7.09	6.93	7.04	7.04	6.87
Glycine	1.64	1.59	1.50	1.53	1.51	1.46
Histidine	0.97	0.96	0.96	0.99	1.00	0.99
Hydroxylysine	0.01	0.05	0.05	0.04	0.03	0.03
Hydroxyproline	0.12	0.10	0.06	0.05	0.04	0.04
Isoleucine	1.74	1.69	1.67	1.68	1.64	1.59
Leucine	3.44	3.26	3.28	3.38	3.35	3.28
Lysine	2.04	2.03	1.97	2.00	1.95	1.89
Methionine	0.62	0.60	0.62	0.61	0.62	0.60
Phenylalanine	1.93	1.86	1.85	1.88	1.85	1.80
Proline	2.32	2.23	2.23	2.30	2.33	2.30
Serine	1.58	1.58	1.56	1.61	1.65	1.63
Taurine	0.23	0.23	0.19	0.21	0.19	0.18
Threonine	1.34	1.33	1.31	1.36	1.36	1.34
Tryptophan	0.48	0.46	0.47	0.45	0.47	0.46
Tyrosine	1.31	1.27	1.25	1.33	1.33	1.30
Valine	1.86	1.81	1.80	1.84	1.83	1.79
Total	37.44	36.38	35.76	36.55	36.41	35.55

*Note:* Shrimp were fed varying inclusion levels of corn fermented protein (CFP), maintained at a stocking density of 30 individuals per tank (~35 shrimp/m^2^), with an initial mean weight of 0.216 ± 0.007 g (mean ± standard deviation). CFP, corn fermented protein (The Andersons, Inc., Maumee, OH, USA).

### 2.3. System and Water Quality

Water quality in the green water trial was sustained through recirculation via two vertical fluidized bed biological filters equipped with Kaldness media. Dissolved oxygen levels were maintained near saturation using two air stones per culture tank and standard airline tubing connected to a regenerative blower. Source water was obtained from an outdoor shrimp production pond via a 1/4 horsepower Baldor Reliance (ABB Motors and Mechanical Inc., Fort Smith, AR, USA) centrifugal sump pump. Daily water exchanges of 5% were conducted to preserve natural productivity within the system.

Dissolved oxygen, temperature, pH, and salinity parameters were monitored twice daily throughout the experimental period, during morning (0700–0715) and afternoon (1500–1515) intervals using a YSI Professional Plus Meter (Yellow Springs Instrument Co., Yellow Springs, OH, USA). Water samples were collected each Monday and Thursday morning. Total ammonia nitrogen (TAN) analysis was performed weekly on Mondays using an ion‐selective electrode, the Orion 4‐Star Plus pH/ISE (Thermo Fisher Scientific, Waltham, MA, USA). On Thursdays, a spectrophotometer, WaterLink Spin Touch FF (LaMotte, Chestertown, MD, USA), was employed to analyze pH, ammonia, nitrite, nitrate, alkalinity, calcium, phosphate, and magnesium. Ammonia values from the WaterLink Spin Touch were converted to TAN following manufacturer protocols. Final TAN values were determined by calculating the mean of ammonia‐N measurements from both the ion electrode and WaterLink Spin Touch systems.

All water parameters reported as mean ± standard deviation (Table 4), namely, dissolved oxygen (6.98 ± 0.49 mg/L), temperature (30.34 ± 2.15°C), salinity (12.25 ± 1.94 g/L), TAN (0.14 ± 0.34 mg/L), and nitrite nitrogen (0.00 ± 0.01 mg/L) were maintained within the suitable range for the development of the cultured animal [23] and were typical for these types of systems.

**Table 4:** Water quality parameters for Pacific white shrimp (*Litopenaeus vannamei*) reared in green water recirculating systems over an 8‐week period.

Parameters	Values
Dissolved oxygen (mg/L)	6.98 ± 0.48 (5.79–7.86)
Temperature (°C)	30.34 ± 2.15 (25.40–33.45)
Salinity (g/L)	12.25 ± 1.94 (9.02–16.15)
Total ammonia nitrogen (mg/L)^a^	0.14 ± 0.34 (0–0.96)
Nitrite nitrogen (mg/L)	0.00 ± 0.01 (0–0.10)
Nitrate nitrogen (mg/L)	0.68 ± 1.58 (0–20.00)
pH	7.94 ± 0.36 (7.90–9.04)
Alkalinity (mg/L)	84.75 ± 17.19 (63.00–111.00)
Calcium (mg/L)	170.50 ± 48.80 (126.00–264.00)
Magnesium (mg/L)	369.25 ± 131.36 (147.00–563.00)
Phosphate (mg/L)	2.99 ± 0.88 (2.10–4.60)

*Note*: Shrimp were fed varying inclusion levels of corn fermented protein (CFP) formulated on an iso‐nitrogenous and iso‐lipidic basis (35 g/100 g protein and 8 g/100 g lipid), maintained at a stocking density of 30 individuals per tank (~35 shrimp/m^2^), with an initial mean weight of 0.216 ± 0.007 g (mean ± standard deviation). Data are presented as mean ± standard deviation, with minimum and maximum values for each parameter shown in parentheses.

^a^Ammonia nitrogen concentrations represent average values measured via ion electrode and WaterLink Spin Touch.

### 2.4. Growth Trial

An 8‐week green water trial was performed at Claude Peteet Mariculture Center, Gulf Shores, AL. Thirty shrimp weighing 0.216 ± 0.007 g (mean ± SD) were randomly distributed into an outdoor green‐water recirculation system comprising 24 tanks (800 L, ~1 m^−2^ bottom surface area with a stocking density of ~35 shrimp/m^2^). The system included two shared reservoir tanks (800 L). Twenty‐four tanks were randomly allocated to six experimental diets, with four tanks serving as replicates for each treatment. Feed was provided four times daily between 0700 and 1500 h.

Biweekly growth monitoring involved bulk‐weighing a subsample of 10 shrimp from one tank per treatment, with this data utilized to modify feeding rates. Daily feed rations were determined based on historical data from prior studies and sampling results, assuming a feed conversion ratio (FCR) of 1.2 and projected weekly weight gains ranging from 0.29 g/week initially to 2.8 g/week at trial completion.

Upon trial conclusion, shrimp were counted and bulk‐weighed. Four randomly chosen shrimp per tank were collected and stored at −20°C for proximate and mineral analyses. An additional four shrimp were euthanized in slurry ice at ~ 0°C and sampled for hemolymph collection, with three of these subsequently dissected to obtain whole hepatopancreases and abdominal intestines (1st to 5th segments), which were preserved in 800 and 300 μL of RNA Shield (ZYMO Research, Irvine, CA, USA) in 5 mL containers and 1.5 mL microcentrifuge tubes, respectively, for gene expression analysis.

Frozen shrimp samples were thawed, cut, weighed, and dried for 24 h at 95°C using a Cole‐Parmer OVF‐400 Series Mechanical Convection Drying Oven (Vernon Hills, IL, USA). The shrimp samples were then pulverized, packaged, and submitted to Midwest Laboratories Inc. (Omaha, NE, USA) for proximate and mineral analysis. When the results were in feed utilization efficiency, including FCR, feed apparent net protein retention (ANPR), and phosphorus retention (PR), were calculated. All metrics were calculated according to Nguyen et al. [24].

### 2.5. Hemolymph Biochemical Analysis

**Figure 1:**
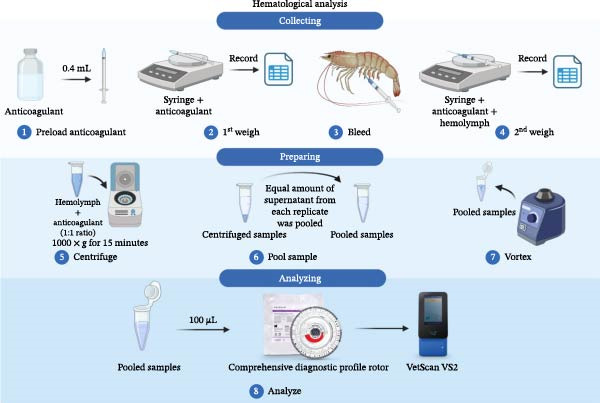
Hemolymph analysis protocol of Pacific white shrimp (*Litopenaeus vannamei*; *n* = 4) cultured in green water recirculation system, fed corn fermented protein at various levels, based on iso‐nitrogenous and iso‐lipidic (35 g/100 g protein and 8 g/100 g lipid) basis during an 8‐week period, stocked at 30 shrimp per tank (~35 shrimp/m^2^) with an initial weight at 0.022 ± 0.007 g (mean ± standard deviation). This image was created with BioRender.com (http://biorender.com/).

Anticoagulant solution was prepared by dissolving 30 mM Trisodium Citrate Dihydrate (Fisher Scientific International L.L.C, Waltham, MA, US), 0.34 M sodium chloride (Fisher Scientific International L.L.C, Waltham, MA, US), 10 mM ethylene diamine tetra acetic acid (EDTA; Sigma–Aldrich Co. Ltd., St. Louis, USA) in de‐ionized (DI) water [25]. Empty and preloaded syringes (BD, Becton, Dickinson, and Company, Franklin Lakes, NJ, USA) with 0.4 μL anticoagulant’s weight was recorded, 0.4 μL hemolymph were collected using a 25‐gauge needle and 1‐cc syringe inserted beneath the carapace via the hemocoel at the cephalothorax–abdominal conjunction shrimp were then bled 0.4 mL to attain a roughly 1:1 ratio between hemolymph and anticoagulant. Afterward, syringes with both anticoagulant and hemolymph were weighed to calculate the dilution factor. All hemolymph and anticoagulant were transferred into a 1.5 mL microcentrifuge tube and subjected to centrifugation at 1000× *g* for 15 min. After centrifugation, an equal amount of supernatant from each replicate tank was pooled into one 1.5 mL microcentrifuge tube. All pooled samples were subjected to mixing before 100 μL of the sample was loaded into a Comprehensive Diagnostic Profile rotor (Zoetis, Union City, CA, USA) and ran on the Abaxis VetScan VS2 (Zoetis, Union City, CA, USA) for alkaline phosphatase (ALP), alanine aminotransferase (ALT), amylase (AMY), calcium, glucose, potassium, and total protein. The returned values from the Vetscan VS2 were then multiplied by the dilution factor of approximately 2.18 ± 0.19 to achieve the final data (Figure 1).

Dilution factor=Weight of anticoagulant+weight of haemolymphWeight of haemolymph.

### 2.6. qPCR Gene Expression Analysis

Intestine and hepatopancreases samples were transferred into a 96‐deep well plate containing 0.1 mm ceramic beads (Omni International, Kennesaw, GA, USA), then, homogenized using Bead Ruptor 96 (Omni International, Kennesaw, GA, USA) at 30 Hz for a total of 20 min, alternating between 5 min of homogenization and 5 min of rest. A total of 100 μL of homogenized samples of the intestine and hepatopancreases samples were extracted and purified using MagMAX *mir*Vana Total RNA Isolation Kit (Thermo Fisher Scientific, Waltham, MA, USA). Sample concentration and quality were measured using a BioTek Take3 Trio microvolume spectrophotometer (Agilent, Santa Clara, CA, USA) to ensure the 260/280 and 260/230 ratios above 1.8. Extracted RNA samples were then diluted and standardized to 40 and 30 ng/μL for intestine and hepatopancreases, respectively. RNA samples were stored at −80°C in an ultralow‐temperature freezer prior to reverse transcription. The conversion to complementary DNA was performed using the High‐Capacity cDNA Reverse Transcription Kit from Applied Biosystems (Waltham, MA, USA) following the manufacturer’s protocol. Each 20 μL reverse transcription reaction contained the following components: 10x R.T. buffer (2 μL), 25x dNTP Mix (0.8 μL), 10x R.T random primers (2 μL), MultiScribe reverse transcriptase (1 μL), nuclease‐free water (4.2 μL), and diluted RNA template (10 μL). Reverse transcription was carried out in a Bio‐Rad T100 thermal cycler (Bio‐Rad Inc., Hercules, CA, USA) using the following temperature profile: initial incubation at 25°C for 10 min, followed by reverse transcription at 37°C for 120 min, and final enzyme inactivation at 85°C for 5 min. Following the reaction, the cDNA samples were diluted to reach a target concentration of 1.25 ng/μL. To ensure the reference gene expression remained consistent across various conditions, reference gene stability was evaluated using NormFinder [26], elongation factor 1 α (*ef1α*) and *l21* were selected as reference genes due to the lowest stability score at 0.04 and 0.11 for intestine and hepatopancreases, respectively. The experiment utilized four target genes: tumor necrosis factor alpha (*tnf-α*), transforming growth factor beta 1 (*tgf-β1*), prophenoloxidase (*propo*), and superoxide dismutaste (*sod*), trypsin (*trypsin*), and chymotrypsin (*chymotrypsin*) with two reference genes *ef1α* and ribosomal protein (*l21*; Table 5). To validate primer performance, efficiency calculations were performed using slope analysis from a five‐point serial dilution series (twofold dilutions) of pooled cDNA derived from 10 samples. Amplification efficiency for each target gene was confirmed to fall within the acceptable range of 90%–110%. Primer efficiency values were computed using the following equation:

**Table 5:** Primers utilized for real‐time qPCR analysis to evaluate physiological and digestive gene expression in Pacific white shrimp (*Litopenaeus vannamei*) reared in green water recirculating systems over an 8‐week period.

Gene	Forward primer (5^’^ to 3^’^)	Reverse primer (5^’^ to 3^’^)	Efficiency (%)	Reference
Cytokines				
* tnf-α*	CTCAGCCATCTCCTTCTTG	TGTTCTCCTCGTTCTTCAC	102.7	[27]
* tgf-β1*	AACCATGCCCTTGTGCAAAC	CTTTGGGGGAACCTCGGTC	104.3	[28]
Immunes				
* propo*	TACATGCACCAGCAAATTATCG	AGTTTGGGGAAGTAGCCGTC	107.7	[29]
* sod*	GCAATGAATGCCCTTCTACC	CAGAGCCTTTCACTCCAACG	101.6	[30]
Digestive				
* trypsin*	TCCAAGATCATCCAACACGA	GACCCTGAGCGGGAATATC	105.2	[31]
* chymotrypsin*	GGCTCTCTTCATCGACG	CGTGAGTGAAGAAGTCGG	108.5	[31]
Reference gene				
* l21*	GTTGACTTGAAGGGCAAT G	CTTCTTGGCTTCGATTCTG	98.6	[32]
* ef1*α	GTATTGGAACAGTGCCCGTG	TCACCAGGGACAGCCTCAGTA	100.3	[33]

*Note*: Shrimp were fed varying inclusion levels of corn fermented protein (CFP) formulated on an iso‐nitrogenous and iso‐lipidic basis (35 g/100 g protein and 8 g/100 g lipid), maintained at a stocking density of 30 individuals per tank (~35 shrimp/m^2^), with an initial mean weight of 0.216 ± 0.007 g (mean ± standard deviation).

Efficiency %=10−1Slope−1×100.

Six microliters of SsoAdvanced Universal SYBR Green Supermix (Bio‐Rad, Hercules, CA, USA), 0.25 μL of each forward and reverse primer (stock concentration of 10 μM), 0.5 μL of nuclease‐free water, and 4 μL of diluted cDNA sample were used in each 10 μL reaction. Each sample was analyzed in duplicate, along with a negative control (nuclease‐free water in place of a cDNA template).

Real‐time PCR amplification was performed using a Bio‐Rad CFX Opus 384 Real‐Time PCR System (Bio‐Rad Inc., Hercules, CA, USA). The thermal cycling protocol consisted of an initial denaturation at 50°C for 2 min and 95°C for 2 min, followed by 40 amplification cycles at 95°C for 15 s, 58°C for 15 s, and 72°C for 30 s. For relative quantification analysis, data were normalized to the geometric mean of two reference genes, *ef1a* and *l21*. Normalized expression values were subsequently expressed relative to the basal treatment group, which was assigned a value of 1, using the 2^−*ΔΔ*Ct^ method [34, 35].

### 2.7. Statistical Analysis

Data analysis and visualization were conducted using R version 4.3.2 [36] and RStudio 2023.12.1.402 [37]. The analysis employed several R packages including tidyverse [38], gtsummary [39], emmeans [40], multcomp [41], factoextra [42], effectsize [43], segmented [44], and ggbiplot [45]. Log_10_ transformation was applied to relative gene expression data to satisfy normality assumptions [46]. Outliers were identified and assessed through box‐whisker plots and Cook’s distance analysis, with the median quantile distribution serving as the threshold for detection [47–49]. Normality of residuals for parametric data was evaluated using the Shapiro–Wilk test, while homogeneity of variances was assessed via Bartlett’s test [50, 51]. Statistical comparisons between parametric means were performed using one‐way analysis of variance (ANOVA), with post hoc pairwise comparisons conducted using Tukey’s honest significant difference test when significant differences for ANOVA were detected. Linear, quadratic, and broken‐line regression models were evaluated for all parameters using the coefficient of determination, Akaike Information Criterion (AICc) corrected, weighted AIC corrected, relative likelihood, and evidence ratio (Table 6) [52]. Based on the model evaluation, the coefficient of determination and *p*‐value of the linear regression model were reported alongside the one‐way ANOVA, unless otherwise mentioned. The relationship between inclusion levels of CFP and shrimp weight, weight gain, PR, and hemolymph calcium were plotted with 95% confidence intervals (CIs). A predetermined significance level of *a* = 0.05 was established for all statistical tests.

**Table 6:** Regression model selection employed to evaluate the relationship between varying inclusion levels of corn fermented protein (CFP) and growth metrics in Pacific white shrimp (*Litopenaeus vannamei*) reared in green water recirculating systems over an 8‐week period.

Parameters	Models	*R* ^2^	Akaike information criterion corrected	Weighted akaike information criterion corrected	Relative likelihood	Evidence ratio
Final weight (g)	Linear	**0.222**	**38.051**	**0.581**	**1.000**	**1.000**
	Quadratic	0.227	39.888	0.232	0.399	2.505
	Broken‐line	—	41.559	0.101	0.173	5.777
Weight gain (%)	Linear	**0.080**	**355.986**	**0.574**	**1.000**	**1.000**
	Quadratic	0.089	357.747	0.238	0.415	2.412
	Broken‐line	—	359.563	0.096	0.167	5.981
Phosphorus retention (%)	Linear	0.206	107.133	0.331	0.026	0.791
	Quadratic	0.2717	107.693	0.250	0.036	0.598
	Broken‐line	**—**	**106.665**	**0.419**	**1.000**	**1.000**
Calcium (mg/dL)	Linear	0.001	121.146	0.228	0.560	1.787
	Quadratic	0.139	120.200	0.365	0.898	1.114
	Broken‐line	**—**	**119.985**	**0.407**	**1.000**	**1.000**

*Note*: Shrimp were fed corn fermented protein (CFP) formulated on an iso‐nitrogenous and iso‐lipidic basis (35 g/100 g protein and 8 g/100 g lipid), maintained at a stocking density of 30 individuals per tank (~35 shrimp/m^2^), with an initial mean weight of 0.216 ± 0.007 g (mean ± standard deviation). Model evaluation was conducted using *R* ^2^, akaike information criterion corrected, akaike information criterion weight corrected, relative likelihood, and evidence ratio values. The bold values indicates the selected model.

Standardized effect sizes, including omega squared (*ω* ^2^) and Hedges’ *g*, were reported to adjust for small sample sizes bias, allowing comparison across studies regardless of measured units. Bias‐corrected omega squared was used to assess the proportion of variance in the dependent variable accounted for by treatment groups utilizing ANOVA, with a 95% CI calculated by using the noncentral parameter (NCP) method. Negative omega squared with 95% were set to zero [53, 54]. Hedges’ *g* effect size was computed with bias‐corrected 95% CI utilizing pooled standard deviations for pairwise comparisons of treatment groups relative to the basal group [55]. Forest plots were created to illustrate Hedges’ *g* values and associated CIs, together with the absolute mean difference. Omega squared and Hedges’ *g* were calculated as follows:

To facilitate cross‐study comparisons independent of measurement units and to correct for small sample bias, standardized effect sizes were calculated, specifically omega squared (*ω* ^2^) and Hedges’ *g*. The proportion of dependent variable variance explained by treatment groups was quantified using bias‐corrected omega squared through ANOVA [56]. CIs (95% CI) for omega squared were determined using the NCP approach, with any negative omega squared values and their 95% CI constrained to zero [54, 56]. For pairwise treatment comparisons against the basal group, Hedges’ *g* was calculated using pooled standard deviations, incorporating bias correction and 95% CI [55]. Visual representation of Hedges’ *g* values, CIs, and absolute mean differences was provided through forest plots. Both omega squared and Hedges’ *g* calculations followed the methodology described by Nguyen et al. [24].

Variation among groups was calculated through the pooled standard error (PSE; [24, 57]), incorporating within‐group variability and average sample size per group

Principal component analysis (PCA) was employed for further examination of gene expression data. Prior to analysis, gene expression values were subjected to standardization through centering and scaling procedures, resulting in variables with zero mean and unit standard deviation. This normalization process ensured equal contribution from all genes during analysis, eliminating bias from differences in original expression magnitudes. The PCA was performed using a covariance matrix approach, which preserves linear associations between variables, while emphasizing variance patterns across genes rather than absolute expression values, thereby enhancing pattern recognition and sample clustering [58]. The proportion of variance attributable to each principal component was displayed through scree plot visualization, with the two components explaining the greatest variance (PC1 and PC2) selected for biplot construction [59]. To illustrate group distribution and variability, confidence ellipses at the 95% level were incorporated into the biplots. Additionally, squared cosine values (cos^2^) were calculated and displayed to evaluate how well individual genes were represented within the PC1 and PC2 dimensions [58].

## 3. Results

### 3.1. Growth Performance and Feed Utilization Efficiency

At the end of the 8‐week trial, growth performances of *L. vannamei* fed different protein sources showed no statistically significant differences amongst diets (*p*>0.05) for all measured parameters (Table 7). No significant differences in average final weight, weight gain, TGC, weekly gain, and FCR were observed in basal diet and diets that contained up to 25% of CFP (*p*>0.05, *ω* ^2^ = 0–0.136). In specific, final average weight reduced from 18.53 g in the basal diet to 17.86 g diet that had 25% of CFP (*p* = 0.117, *ω* ^2^ = 0.117, 95% CI: 0.000, 0.265), while that of FCR went up from 1.09 with no inclusion rate to 1.12 at highest inclusion rate (*p* = 0.372, *ω* ^2^ = 0.000, 95% CI: 0.000, 0.000). In line with the previous outcome, the TGC indicated a steady decrease moving toward 25% inclusion of CFP; however, this outcome was not statistically significant (*p* = 0.215, *ω* ^2^ = 0.108, 95% CI: 0.000, 0.244). Linear regression showed 16.9%–27.7% variation in final weight (Figure 2A), weekly gain, TGC, and FCR explained the experimental diets (*p*<0.05). In contrast, inclusion levels of CFP explained only 9.8% of the variation in weight gain percent (*p* = 0.136; Figure 2B). Despite showing no significant difference, Hedges’ *g* effect size for final weight confirmed that the higher the inclusion rate of CFP, the higher the absolute difference relative to basal diet was seen, with effect size ranging between −0.076 and 1.176 (Figure 3A). In concurrence, the FCR reflected slightly higher values with a higher CFP inclusion rate (Figure 3B).

Figure 2Relationship between inclusion levels of CFP and performance metrics of Pacific white shrimp (*Litopenaeus vannamei*) cultured in green water recirculating system fed various levels of CFP: Basal (*n* = 4), CFP 5% (*n* = 4), CFP 10% (*n* = 4), CFP 15% (*n* = 4), CFP 20% (*n* = 4), and CFP 25% (*n* = 4) based on iso‐nitrogenous and iso‐lipidic (35 g/100 g protein and 8 g/100 g lipid) basis during an 8‐week period, stocked at 30 shrimp per tank (~35 shrimp/m^2^) with an initial weight at 0.022 ± 0.007 g (mean ± standard deviation). (A) Final weight (g), (B) weight gain (%), (C) phosphorus retention (%), and (D) hemolymph calcium levels (mg/dL). Graphs were constructed based on linear and segmented regression with a 95% confidence interval.

**Figure:**
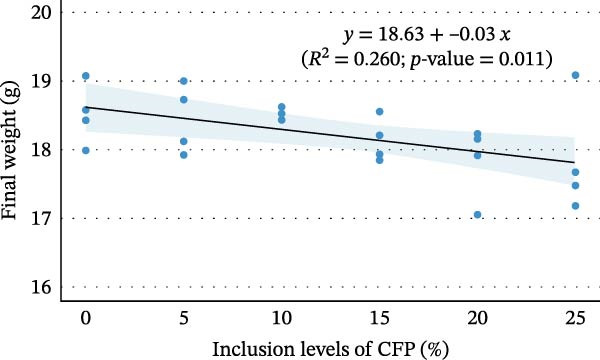
(A)

**Figure:**
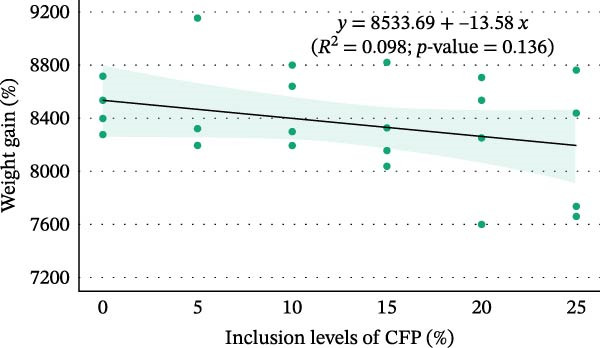
(B)

**Figure:**
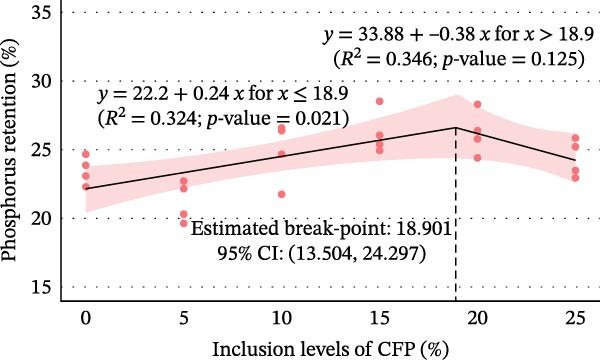
(C)

**Figure:**
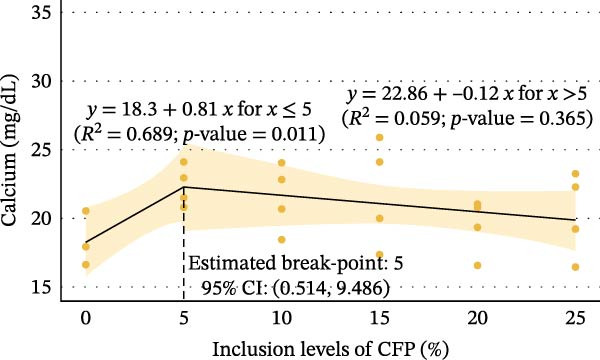
(D)

Figure 3Hedges’ *g* effect size of shrimp performance metrics in greenwater recirculating system. (A) Final weight, (B) feed conversion ratio, and (C) apparent net protein retention of shrimp fed various level of CFP: CFP 5% (*n* = 4), CFP 10% (*n* = 4), CFP 15% (*n* = 4), CFP 20% (*n* = 4), and CFP 25% (*n* = 4) in comparison to the basal diet (*n* = 4). Each panel includes absolute mean differences, forest plots illustrating Hedges’ *g* effect sizes with confidence intervals, and classifications of effect significance: significant increase (green), nonsignificant (gray), or significant decrease (red). Positive effect sizes indicate improved performance relative to the basal diet, while negative values indicate reduced performance.

**Figure:**
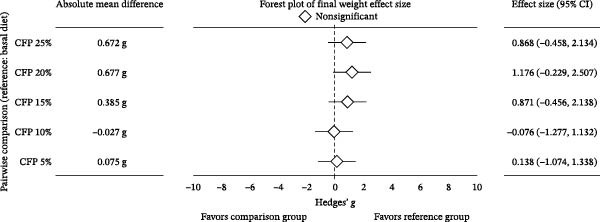
(A)

**Figure:**
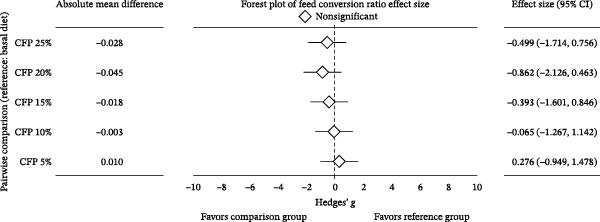
(B)

**Figure:**
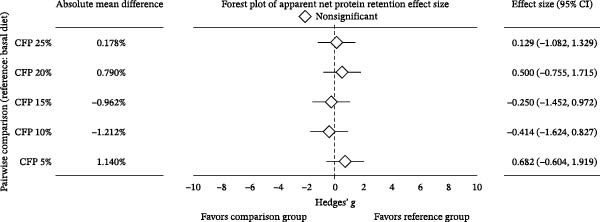
(C)

**Table 7:** Growth performance and feed utilization of Pacific white shrimp (*Litopenaeus vannamei*) reared in green water recirculating systems over an 8‐week period.

Diet	Survival (%)	Final weight (g)	Weight gain (%)	Weekly gain (g)	Thermal‐unit growth coefficient	Feed conversion ratio	Apparent net protein retention (%)	Phosphorus retention (%)
Basal	98.35	18.53	8481	2.29	3.59	1.09	44.86	23.53^ab^
CFP 5%	98.35	18.46	8464	2.28	3.58	1.08	43.72	21.23^b^
CFP 10%	99.18	18.56	8480	2.29	3.60	1.09	46.07	24.90^a^
CFP 15%	98.35	18.15	8334	2.24	3.52	1.11	45.82	26.25^a^
CFP 20%	96.68	17.86	8273	2.20	3.46	1.13	44.07	26.26^a^
CFP 25%	100.00	17.86	8149	2.21	3.46	1.12	44.68	24.35^ab^
One‐way ANOVA								
PSE	0.94	0.26	201	0.03	0.05	0.02	1.34	0.793
*ω* ^2^ (95% CI)	0.075 (0.000, 0.154)	0.117 (0.000, 0.264)	0.000 (0.000, 0.000)	0.136 (0.000, 0.301)	0.108 (0.000, 0.244)	0.000 (0.000, 0.000)	0.000 (0.000, 0.000)	0.494 (0.000, 0.680)
* p*‐Value	0.275	0.201	0.804	0.172	0.215	0.372	0.784	0.003
Regression								
*R* ^2^	0.004	0.260	0.098	0.277	0.254	0.169	2.98e–06	—^1^
* p*‐Value	0.776	0.011	0.136	0.008	0.012	0.046	0.994	—^1^

*Note:* Values represent the mean of four replicates of each diet. Means not sharing any letter are significantly different by the Tukey’s HSD‐test (parametric ANOVA) at the 5% level of significance. Shrimp were fed varying inclusion levels of corn fermented protein (CFP) formulated on an iso‐nitrogenous and iso‐lipidic basis (35 g/100 g protein and 8 g/100 g lipid), maintained at a stocking density of 30 individuals per tank (~35 shrimp/m^2^), with an initial mean weight of 0.216 ± 0.007 g (mean ± standard deviation). CFP, corn fermented protein (The Andersons, Inc., Maumee, OH, USA)

^1^Broken‐line regression model was selected.

Abbreviation: PSE pooled standard error.

In terms of feed consumption efficiency, an identical trend was seen, with no statistically significant difference in ANPR (*p* = 0.784, *ω* ^2^ = 0.000, 95% CI: 0.000, 0.000), except for PR (*p* = 0.003, *ω* ^2^ = 0.494, 95% CI: 0.000, 0.680). Specifically, ANPR across shrimp groups shared a similar range from 43.72% to 46.07%. Shrimp fed CFP at 5% for PR appeared to have lower retention compared to 15% and 20% inclusion rates. However, no noteworthy difference existed between the highest inclusion rate and the basal diet. Linear regression analyses indicated that the diet had a negligible effect on ANPR, accounting for less than 0.05% (*p* = 0.994). For PR, segmented regression revealed that at 18.90% (95% CI: 13.5, 24.30) or less, for each 1% increase in CFP in the diet, shrimp retained 0.24% more in phosphorus (*R* ^2^ = 0.324, *p* = 0.021). Despite not showing statistically significant, the break‐point at 18.90% marked a shift in which, above this range, PR in shrimp started to decrease at 0.38% for every 1% addition of CFP in shrimp diet (*R* ^2^ = 0.346, *p* = 0.125; Figure 2C). A pairwise comparison for Hedges’ *g* illustrated the minimal impact of CFP compared to basal‐fed shrimp, with CIs that crossed zero (Figure 3C).

### 3.2. Whole‐Body Composition

The whole‐body composition showed no noticeable difference across CP, crude fat, ash, phosphorus, copper, and zinc (Table 8). Despite no statistical significance, there was an observable trend in crude fat in which shrimp accumulated more fat with more CFP consumed, ranging from 7.78% to 8.43%. The linear regression model captured less than 8.7% of the effect of diets on ash, CP, crude fat, phosphorus, and copper in shrimp bodies (*p*>0.05). Similarly, the quadratic regression model illustrated 23.9% of variation attributed to diets for moisture and dry matter content (*p* = 0.057).

**Table 8:** Whole‐body proximate composition (g/100 g dry weight) of Pacific white shrimp (*Litopenaeus vannamei*) reared in green water recirculating systems over an 8‐week period.

Diet	Moisture (%)^a^	Dry matter (%)^a^	Ash (%)	Crude protein (%)	Crude fat (%)	Phosphorus (%)	Copper (%)
Basal	74.40	25.60	11.75	72.83	7.78	1.31	67.88
CFP 5%	74.70	25.30	11.93	72.08	7.53	1.30	66.65
CFP 10%	74.44	25.56	11.23	72.65	7.98	1.36	66.80
CFP 15%	74.69	25.31	12.00	73.20	7.57	1.30	66.95
CFP 20%	74.87	25.13	12.05	72.53	8.11	1.31	68.88
CFP 25%	75.47	24.53	11.83	72.28	8.43	1.32	67.33
One‐way ANOVA							
PSE	0.674	0.674	0.212	0.463	0.487	0.021	2.30
*ω* ^2^ (95% CI)	0.000 (0.000, 0.000)	0.000 (0.000, 0.000)	0.000 (0.000, 0.000)	0.000 (0.000, 0.000)	0.000 (0.000, 0.000)	0.000 (0.000, 0.000)	0.000 (0.000, 0.000)
* p*‐Value	0.886	0.886	0.124	0.599	0.774	0.345	0.982
Regression							
*R* ^2^	0.239^b^	0.239^b^	0.026	0.002	0.087	0.007	0.002
* p*‐Value	0.057^b^	0.057^b^	0.448	0.827	0.243	0.691	0.820

*Note:* Values represent the mean of four replicates of each diet. Means not sharing any letter are significantly different by the Tukey’s HSD‐test (Parametric ANOVA) at the 5% level of significance. Shrimp were fed varying inclusion levels of corn fermented protein (CFP) formulated on an iso‐nitrogenous and iso‐lipidic basis (35 g/100 g protein and 8 g/100 g lipid), maintained at a stocking density of 30 individuals per tank (~35 shrimp m^−2^), with an initial mean weight of 0.216 ± 0.007 g (mean ± standard deviation). CFP, corn fermented protein (The Andersons, Inc., Maumee, OH, USA).

^a^Based on an as is basis.

^b^Quadratic regression model was selected.

Abbreviation: PSE, pooled standard error.

### 3.3. Hemolymph Analysis

With respect to hemolymph parameters, shrimp fed various levels of diets containing CFP yielded no significant difference between groups (*p*>0.05; Table 9). In specific, enzymatic activity of concern, ALT, and AMY varied between shrimp groups with point estimate for omega squared ranging from 0 to 0.06. Calcium, glucose, potassium, and total protein levels unveiled little to no statistical impact on CFP (*p*>0.05, *ω* ^2^ = 0.000–0.087). Linear regression models demonstrated similar outcomes in which the coefficient of determination ranged from less than 2% to 12.8% across hemolymph parameters with *p*  > 0.05. For calcium, the segmented regression model illustrated that when reaching above 5% (95%: 0.51, 9.48) of CFP in shrimp diet, each percent increase in CFP would result in 0.12 mg/dL decrease in calcium level (*R* ^2^ = 0.059, *p* = 0.365).

**Table 9:** Hemolymph parameters of Pacific white shrimp (*Litopenaeus vannamei*) reared in green water recirculating systems over an 8‐week period.

Diet	Alkaline phosphatase (U/L)	Alanine aminotransferase (U/L)	Amylase (U/L)	Calcium (mg/dL)	Glucose (mg/dL)	Potassium (mmol/L)	Total protein (g/dL)
Basal	31.30	259	402	18.30	34.86	13.80	11.29
CFP 5%	20.39	272	452	22.34	37.20	13.98	12.10
CFP 10%	25.41	301	428	21.53	47.77	12.64	10.77
CFP 15%	24.83	360	460	21.83	45.49	13.71	11.56
CFP 20%	20.11	279	509	19.47	39.32	13.80	11.41
CFP 25%	17.56	262	432	20.35	41.69	13.62	11.17
One‐way ANOVA							
PSE	4.86	63	32	1.28	4.86	0.72	0.67
*ω* ^2^ (95% CI)	0.000 (0.000, 0.000)	0.000 (0.000, 0.000)	0.060 (0.000, 0.096)	0.087 (0.000, 0.191)	0.000 (0.000, 0.000)	0.000 (0.000, 0.000)	0.000 (0.000, 0.000)
* p*‐Value	0.426	0.863	0.306	0.253	0.430	0.811	0.819
Regression							
*R* ^2^	0.128	0.002	0.071	—^a^	0.038	0.0002	0.006
* p*‐Value	0.086	0.851	0.208	—^a^	0.359	0.950	0.730

*Note:* Values represent the mean of four replicates of each diet. Means not sharing any letter are significantly different by the Tukey’s HSD‐test (Parametric ANOVA) at the 5% level of significance. Shrimp were fed varying inclusion levels of corn fermented protein (CFP) formulated on an iso‐nitrogenous and iso‐lipidic basis (35 g/100 g protein and 8 g/100 g lipid), maintained at a stocking density of 30 individuals per tank (~35 shrimp/m^2^), with an initial mean weight of 0.216 ± 0.007 g (mean ± standard deviation). CFP, corn fermented protein (The Andersons, Inc., Maumee, OH, USA).

^a^Broken‐line regression model was selected.

Abbreviation: PSE, pooled standard error.

### 3.4. qPCR Analysis of Gene Expression

Target genes illustrated similar expression across experimental diets with no noticeable expression observed for *tnf-α*, *tgf-β1*, *propo*, *sod*, *trypsin*, and *chymotrypsin* (*p*>0.05; Figure 4). In particular, there was no clear pattern in which pro‐inflammatory cytokine *tnf-α* was upregulated as shrimp were fed a higher inclusion rate of CFP (*p* = 0.543; Figure 4A), while anti‐inflammatory cytokine *tgf-β1* showed similar expression for all shrimp groups (*p*>0.874; Figure 4B). With respect to *propo* and *sod* (Figure 4C,D), no significant up‐ or downregulation could be observed in shrimp diets that had CFP in comparison to basal diets (*p*>0.05). Similarly, digestive‐related genes in the hepatopancreases, *trypsin* and *chymotrypsin*, showed no statistical up‐ or downregulation across shrimp groups fed different diets (*p*>0.05; Figure 4E,F).

Figure 4Gene expression of Pacific white shrimp (*Litopenaeus vannamei*) cultured in green water recirculating system fed various levels of CFP: Basal (*n* = 4), CFP 5% (*n* = 4), CFP 10% (*n* = 4), CFP 15% (*n* = 4), CFP 20% (*n* = 4), and CFP 25% (*n* = 4) based on iso‐nitrogenous and iso‐lipidic (35 g/100 g protein and 8 g/100 g lipid) basis during an 8‐week period, stocked at 30 shrimp per tank (~35 shrimp/m^2^) with an initial weight at 0.022 ± 0.007 g (mean ± standard deviation). Each replicate consisted of two technical replicates in one qPCR reaction. Expression of *tnf-α* (A), *tgf-β1* (B), *propo* (C), and *sod* (D) of 1st to 5th intestinal segment. Expression of *trypsin* (E) and *chymotrypsin* (F) of hepatopancreases. Bar graphs were constructed using relative expression (2^−*ΔΔ*Ct^), while statistical analyses including error bar (mean ± standard error of the mean), *p*‐value, and PSE were performed based on log_10_ transformation of relative expression. CFP, corn fermented protein (The Anderson, Maumee, OH, USA); PSE, pool standard error.

**Figure:**
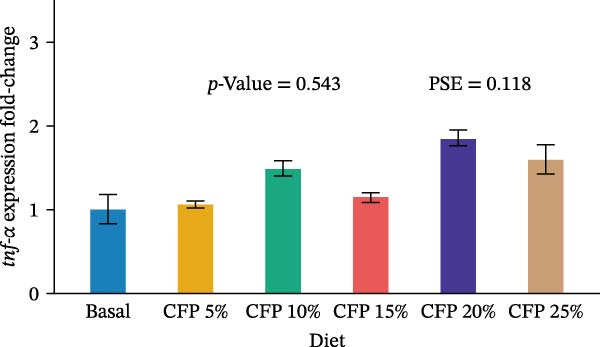
(A)

**Figure:**
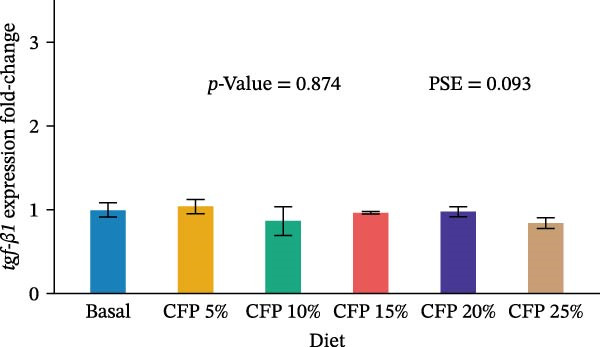
(B)

**Figure:**
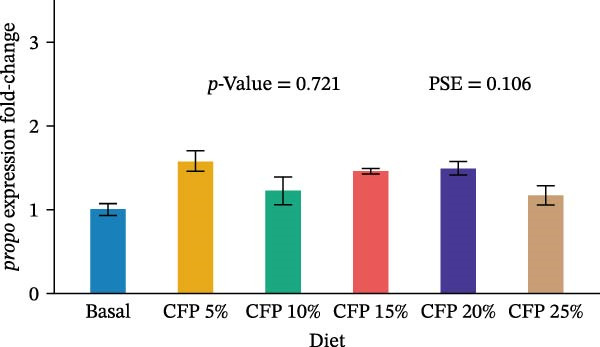
(C)

**Figure:**
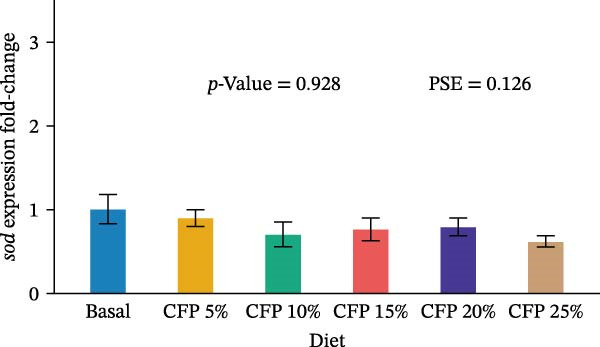
(D)

**Figure:**
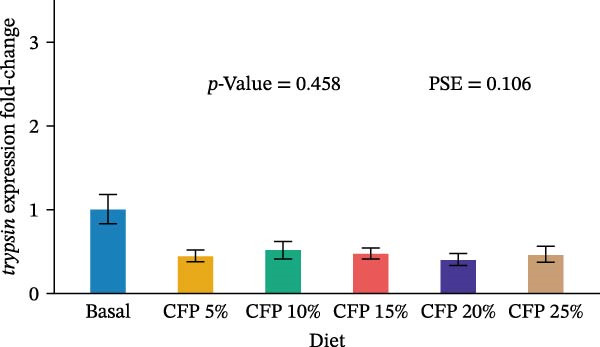
(E)

**Figure:**
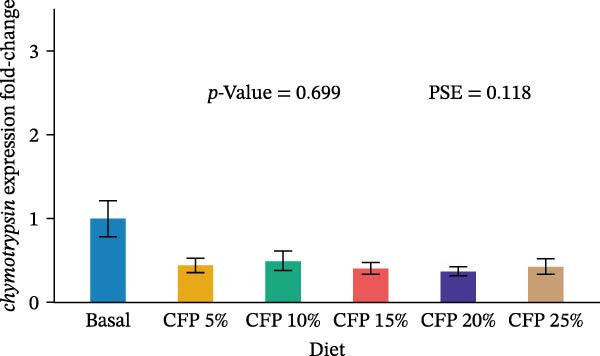
(F)

Principal component 1 explained 56.28% of the total variance, as shown in the scree plot (Figure 5A), demonstrating that it represented the primary driver of gene expression variation across dietary treatments. Principal component 2 explained an additional 21.97%, demonstrating that the first two components together captured 78.25% of the total variance. Analysis of representation quality revealed that *chymotrypsin* and *sod* exhibited the strongest representation, with PC1 and PC2 explaining approximately 4.8% of their variance, establishing these genes as the primary contributors to dietary variation patterns. *tnf-α* and *trypsin* demonstrated moderate influence with contributions of approximately 4.6%, whereas *propo* and *tgf-β1* showed lower contributions at approximately 2.5% and 2.3%, respectively, indicating reduced influence on the overall variation structure (Figure 5B). The biplot showed PC1 was dominated by *propo*, *tnf-α*, *trypsin*, and *chymotrypsin*, while *sod* contributed more to PC2 (Figure 5C). The pattern showed that shrimp fed CFP clustered in the center with similar expression profiles across shrimp groups.

Figure 5Principal component analysis (PCA) of gene expression in Pacific white shrimp (*Litopenaeus vannamei*) cultured in green water recirculating system for eight weeks, fed different protein sources based on iso‐nitrogenous and iso‐lipidic (35 g/100 g protein and 8 g/100 g lipid) basis, stocked at 30 shrimp per tank (~35 shrimp/m^2^) with an initial weight at 0.022 ± 0.007 g (mean ± standard deviation). PCA was constructed using a centered covariance matrix on relative expression (2^−*ΔΔ*Ct^) of *trypsin* and *chymotrypsin* in hepatopancreases, *tnf-α*, *tgf-β1*, *propo*, and *sod* genes in 1st to 5th intestinal segment. CFP, corn fermented protein (The Anderson, Maumee, OH, USA). (A) Eigenvalue screen plot (based on centered covariance matrix). (B) Representation of variables bar plot (based on squared cosine value of PC1 and PC2). (C) Principal component analysis biplot (based on centered covariance matrix).

**Figure:**
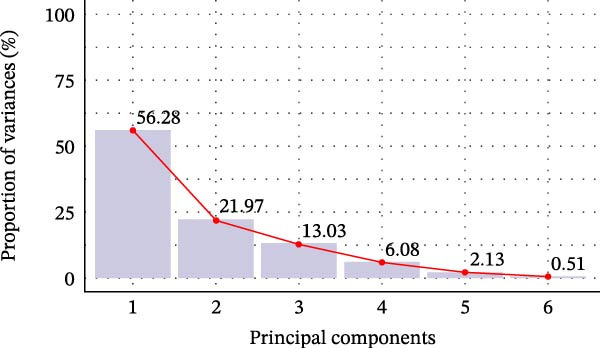
(A)

**Figure:**
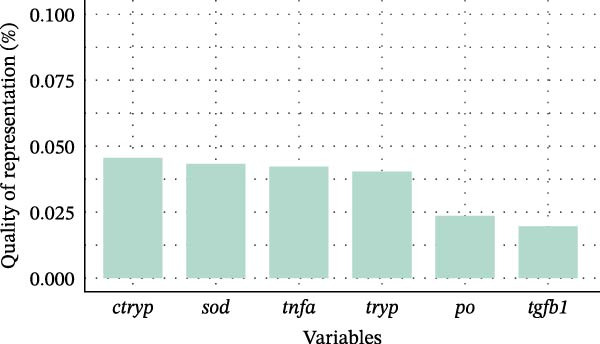
(B)

**Figure:**
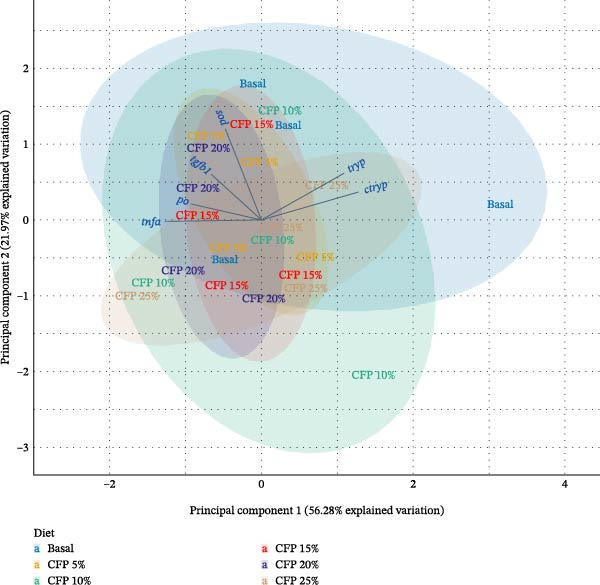
(C)

## 4. Discussion

To fulfill nutritional needs for diet formulation while managing feed costs, there is a need to have a range of protein sources available to allow substitution. Several corn‐based products have been developed, including DDGS, CFP, and CPC, each providing various nutrient profiles and cost variations. Although corn‐derived products possess sufficient protein levels, they often exhibit an unbalanced essential AA (EAA) profile. This study assessed the feasibility of utilizing CFP as a protein source. For this work, we incrementally substituted the three main protein sources‐solvent‐extracted SBM, CPC, and FM in practical shrimp feed formulations. Experimental diets were formulated to maintain equivalent nitrogen and lipid content, with EAA profiles balanced to avoid nutritional deficiencies.

Growth metrics demonstrated no significant impact of CFP inclusion of up to 25% on growth and feed utilization parameters in the presence of natural productivity. A linear regression model of final weight showed a moderate relationship, in which increased CFP in the feed was associated with a greater reduction in growth (Figure 2A). It is worth noting that weight gain percent (Figure 2B), despite showing a similar trend to that of final weight, did not reflect such an influence, with only 9.8% of variation explained by diet effects. In concurrence with these results, previous studies on Pacific white shrimp in clear and green water conditions show that the substitution of FM and CPC to CFP showed no significant influence on growth performance [21, 22]. Similarly, with the effort to reduce CPC in *L. vannamei* feed under clear water conditions, Nazeer et al. [19] observed no noticeable effect of HP‐DDGS and CFP (HP40Y and HP50Y) on growth and feed utilization with up to 20% inclusion rate for both tested ingredients. In contrast, Rhodes et al. [20] demonstrated that substituting SBM with DDGS at up to 10% yielded higher final weight than 20%–40% DDGS. For feed utilization efficiency, PR decreased when the diet contained more than 18.9% of CFP. However, such a trend was not observed in the FCR and ANPR. In fact, FCR and ANPR remained similar across diets, which was similar to those studies of Guo et al. [22] and Nazeer et al. [19]. Additionally, omega squared values of 0.000 (95% CI: 0.000, 0.000) were observed for WG (%), FCR, and ANPR. These values demonstrate that within‐group variation (random effects) substantially exceeded between‐group variation attributable to dietary treatments, rather than indicating a complete absence of dietary influence [54]. Under the reported conditions in which three main protein sources were substituted with CFP, the influence of tested diets on shrimp growth with the presence of natural productivity was at a moderate level, as presented by both effect sizes and the regression model. This gives flexibility to aquaculture nutritionists as well as feed formulators to have well‐balanced shrimp feed while maintaining a reasonable price point, thus reducing production costs.

Crustaceans, particularly shrimp, possess an open circulatory system wherein hemolymph circulates directly through organs within a body cavity termed the hemocoel [60]. This system not only performs the fundamental function of transporting oxygen and nutrients, comparable to vertebrate closed circulatory systems but also performs other roles. It serves as a reservoir for essential metabolic and immunological activities, storing minerals necessary for molting and promoting blood coagulation to prevent pathogen intrusion. Additionally, shrimp hemolymph has enzymes for food decomposition and chemicals that serve as health indicators, reflecting the organism’s physiological state [61]. ALP, among the several enzymes present in shrimp hemolymph, is a crucial component of the humoral immune system, aiding in tissue healing and immunological responses. Additionally, ALT is essential for AA metabolism, reflecting the metabolic and nutritional status of the shrimp [62, 63]. Furthermore, AMY, a crucial digestive enzyme, facilitates the transformation of starches into simpler sugars, improving nutrient absorption and energy availability [64, 65]. The present investigation revealed no effect of differing CFP inclusion amounts on the enzymatic activity of ALP, ALT, and AMY in shrimp plasma. This outcome aligns with the findings of Galkanda‐Arachchige et al. [21], indicating that ALP and ALT levels were comparable among shrimp groups, with no statistically significant differences seen between the basal and the maximum inclusion of CFP in the diet of Pacific white shrimp.

Stress arises when an organism’s equilibrium, or homeostasis, is disturbed by internal or external agents referred to as stressors [66]. Stress initiates a rapid sequence of molecular and physiological changes, which are frequently observable and quantifiable. Amongst stress indicators are glucose and total protein as they function as a marker of metabolic activity and short‐term stress response in crustaceans typically attributed to handling and hypoxia condition [67–72]. In addition, like growth performance metrics, omega squared values of zero should not be interpreted as having no effect from tested diets. The present investigation did not detect any statistical and practical impact of various inclusion levels of CFP, demonstrating no stressful events that triggered the physiological response of Pacific white shrimp.

Marine shrimp, specifically *L. vannamei*, unlike certain other crustaceans, do not possess gastroliths for the storage of vital minerals utilized during molting or blood coagulation [73–75]. Rather, they retain essential minerals such as calcium and potassium inside their hemolymph, where they facilitate these vital functions [76]. Especially calcium ions, which are crucial in blood coagulation, since they activate transglutaminase, an enzyme that stabilizes clots by cross‐linking proteins to provide a protective barrier against infections and tissue damage [77]. Under the experimental conditions, like the previously referenced hemolymph measures, no significant effect of the assessed diets on calcium and potassium levels was detected in Pacific white shrimp grown in a green water system. While calcium levels exhibited a slight decline with dietary CFP inclusion over 5%, this should not be regarded as an adverse consequence of elevated CFP levels. When exposed to suitable salinity levels, marine shrimp may directly absorb vital minerals such as calcium from the water to sustain physiological equilibrium. This process necessitates energy and can trigger physiological reactions similar to stress [68]. The current study showed no stress reactions in enzyme activity as well as glucose and total protein, indicating that a CFP incorporation level of up to 25% is acceptable, with an ideal level of approximately 10%.

Analyzing gene expression in the shrimp gut and hepatopancreas is essential for comprehending the impact of varying inclusion rates of CFP on health and digestive capability. By examining pro‐inflammatory (*tnf-α*), anti‐inflammatory (*tgf-β1*), immune‐related genes (*propo* and *sod*), and digestive enzymes (*trypsin* and *chymotrypsin*) gene expression, the effects of CFP levels can be assessed. TNF‐α, an important inflammatory mediator, improves phagocytosis and macrophage function to counteract infections [78]. The present work indicates that the absence of notable alterations in *tnf-α* regulation implies a lack of inflammation in the shrimp gut when subjected to low FM and various levels of CFP diets. This data contradicts findings from Rahimnejad et al. [28], who noted considerable elevation of *tnf-α* in shrimp consuming a low‐FM diet. TGF‐β1, an anti‐inflammatory cytokine, is involved in the signaling of cell formation, proliferation, and migration in leukocytes, resulting in aiding in the suppression of inflammation [79, 80]. This research found no substantial modulation of *tgf-β1*, possibly owing to the lack of intestinal inflammation, which was similar to the expression profile of *tnf-α*. ProPO, an enzyme that plays a crucial role in the melanization and encapsulation of pathogens, serves as a significant predictor of the shrimp’s capacity to initiate an efficient immune response [81]. No changes in *propo* expression were seen in shrimp when substituting FM, CPC, and SBM diets to CFP, suggesting that immune activity was neither augmented nor diminished [29, 82]. Additionally, SOD, an antioxidant enzyme, neutralizes reactive oxygen species (ROS) to protect shrimp cells against oxidative damage. This enzyme converts harmful superoxide anions into hydrogen peroxide and oxygen, with the resulting hydrogen peroxide subsequently processed by catalase and glutathione peroxidase into water and oxygen [83]. This research indicates that *sod* expression in the gut was relatively similar across shrimp groups, suggesting no clear evidence of severe oxidative stress or immunological disturbance. This corresponds with previous research indicating heightened SOD activity in *L. vannamei* as an indicator of enhanced immunological health [29, 84] trypsin and chymotrypsin belong to the serine protease family and are alkaline proteolytic enzymes in the shrimp hepatopancreas, essential for digestion and immune defense, including activating crustaceans prophenoloxidase [85–87]. Their concentrations are reliant on life stages, enzyme secretion, mRNA transcription synthesis, biochemical adaption, and breakdown inside the digestive system [88–90]. Research indicated that Pacific white shrimp given certain protein levels exhibited an upregulation of trypsin and chymotrypsin relative to those receiving higher or lower protein levels [31, 91]. The present investigation demonstrated a generally reduced expression of trypsin and chymotrypsin in shrimp groups given CFP; however, this tendency lacked statistical significance.

PCA reduces the dimensionality of large datasets, including several variables of interest. Numerous investigations have documented the application of PCA for examining gene expression variation between treatment groups across several fish species, including Atlantic salmon (*Salmo salar*), grass carp (*Ctenopharyngodon idella*), pikeperch (*Sander lucioperca*), and striped catfish (*Pangasianodon hypophthalmus*) [92–95]. The present study used PCA to determine the distribution of genes of interest across various CFP levels. The results demonstrated a distinct pattern whereby all shrimp groups mostly congregated near the center, showing little expression in relation to PC1 or PC2 (Figure 5C). Notably, with a 25% incorporation of CFP, the expression profile of shrimp altered in contrast to other shrimp groups. Nevertheless, the quality of gene representation typically remained below 5% (Figure 5B), indicating that the diets analyzed did not affect the overall variance in gene expression [58]. Based on the aforementioned data, it is justifiable to assert that a CFP content of up to 25% would not adversely affect the health or digestive capacity of Pacific white shrimp. Increasing CFP above 25% while lowering FM, CPC, and solvent‐extracted SBM may provide a possible concern, the specific mechanisms of which remain unknown.

## 5. Conclusion

In conclusion, the biological response of *L. vannamei* was not adversely affected by the incorporation of CFP as a replacement for conventional protein sources, including FM, CPC, and solvent‐extracted SBM, in practical shrimp feed. The results demonstrate that the incorporation of up to 25% CFP in shrimp diets did not significantly impact growth performance, feed efficiency, or hemolymph metrics, including enzymatic activity (ALP, ALT, and AMY) and stress markers (glucose and total protein). Moreover, calcium and potassium concentrations in the hemolymph remained consistent, indicating that shrimp maintained physiological homeostasis despite increased CFP incorporation. Gene expression study indicated no significant changes in immune‐related genes (*tnf-α*, *tgf-β1*, *propo*, and *sod*) or digestive enzyme genes (*trypsin* and *chymotrypsin*), consistent with the negligible effects seen in growth and physiological responses. These results illustrate the potential of CFP as a cost‐efficient and nutritionally sound component in shrimp feed compositions. Nonetheless, CFP inclusion above 25% may provide challenges, and the precise processes require more examination. Ongoing investigation into alternate protein sources and their impacts on shrimp health, performance, and feed efficiency is crucial for optimizing aquaculture techniques.

## Funding

Funding for this work was provided in part through the Alabama Agricultural Station and the Hatch program (Grant ALA016‐1‐19102) administered by the National Institute of Food and Agriculture, U.S. Department of Agriculture.

## Disclosure

Reference to commercial trademarks and proprietary products in this manuscript does not imply endorsement by Auburn University, nor does it suggest exclusion of other potentially suitable products or services.

## Ethics Statement

Animal care protocols were in compliance with Auburn University animal care and use policies.

## Conflicts of Interest

The authors declare no conflicts of interest.

## Data Availability

The data that support the findings of this study are available from the corresponding author upon reasonable request.
